# Rising nutrient-pulse frequency and high UVR strengthen microbial interactions

**DOI:** 10.1038/srep43615

**Published:** 2017-03-02

**Authors:** Marco J. Cabrerizo, Juan Manuel Medina-Sánchez, Irene Dorado-García, Manuel Villar-Argaiz, Presentación Carrillo

**Affiliations:** 1 Departamento de Ecología, Facultad de Ciencias, Universidad de Granada, Campus Fuentenueva s/n, 18071, Granada España; 2 Instituto Universitario de Investigación del Agua, Universidad de Granada, C/Ramón y Cajal, 4, 18071, Granada España

## Abstract

Solar radiation and nutrient pulses regulate the ecosystem’s functioning. However, little is known about how a greater frequency of pulsed nutrients under high ultraviolet radiation (UVR) levels, as expected in the near future, could alter the responses and interaction between primary producers and decomposers. In this report, we demonstrate through a mesocosm study in lake La Caldera (Spain) that a repeated (*press*) compared to a one-time (*pulse*) schedule under UVR prompted higher increases in primary (PP) than in bacterial production (BP) coupled with a replacement of photoautotrophs by mixotrophic nanoflagellates (MNFs). The mechanism underlying these amplified phytoplanktonic responses was a dual control by MNFs on bacteria through the excretion of organic carbon and an increased top-down control by bacterivory. We also show across a 6-year whole-lake study that the changes from photoautotrophs to MNFs were related mainly to the frequency of pulsed nutrients (e.g. desert dust inputs). Our results underscore how an improved understanding of the interaction between chronic and stochastic environmental factors is critical for predicting ongoing changes in ecosystem functioning and its responses to climatically driven changes.

Nutrients and light are two key factors that regulate the ecosystem’s functioning. Nutrient pulses are stochastic events of brief resource availability that have strong effects on a huge variety of ecological processes (e.g. growth, primary production [PP], consumer-resource interaction) in a range of ecosystems worldwide^1^,^2^. Although Yang *et al*.^3^ have clearly defined pulsed resources, a major unresolved question is how the increasing frequency of such events due to global climate change^4^ could alter ecosystem dynamics in the future. Specifically, the heavy transport of atmospheric dust due to severe droughts related to climate change and positive anomalies in the North Atlantic Oscillation Index (+NAO) implies greater mineral inputs to aquatic ecosystems worldwide^5^. These higher mineral inputs to ecosystems may be particularly relevant in the Mediterranean region due to its proximity with the Sahara Desert, which constitutes the largest global dust-export source of mineral nutrients, especially inorganic phosphorus (P)^6^,^7^. These potential increases in the P inputs may be particularly critical in severely nutrient-limited aquatic environments such as open-sea areas^8^,^9^ or oligotrophic high-mountain lakes^10^,^11^, because of the relatively greater exposure to atmospheric deposition and/or the minimal influence of catchment vegetation^12^. Brief events of pulsed nutrients in these ecosystems can account for a large proportion of all available resources, which can have persistent repercussions for primary producers, consumers and decomposers^13^,^14^. These persistent effects result from the altered cell size and structure of the microbial community^15^,^16^ as well as from altered key processes such as PP and trophic interaction within the microbial loop^17^,^18^.

Ever since the pioneering study by Azam *et al*.^19^, information has been progressively amassed concerning the complex interactions between the nutritional–physicochemical environment and the components of the microbial food web in oligotrophic ecosystems. Surges in nutrient availability are known to reduce competition between phytoplankton and bacteria and stimulate their growth^20^. This stimulatory effect on both compartments and the reported increase in the excretion of organic carbon (EOC) by phytoplankton^21^,^22^, can be propagated from the microbial loop^23^,^17^ to the grazing chain^24^. Therefore, the microbial loop development depends on the coupling between phytoplankton and bacteria. This coupling is defined as the capacity of the carbon (C) released by the phytoplankton to support the bacterial C requirement^25^ and the degree of coupling differs depending on the inorganic nutrient availability^26^,^21^, ultraviolet radiation (UVR) exposure^27^, temperature^22^ or stoichiometric balances^28^,^29^. However, this traditional paradigm of food-web structure is being challenged by a current of thought that suggests an alternative new paradigm in which the bulk of the base of this food web is supported by protist planktonic communities that are mixotrophic, combining phagotrophy and phototrophy within the same organism, instead of photoautotrophic phytoplankton^30^. In fact, an increasing number of studies show the prevalence of mixotrophs in natural communities, particularly in surface waters of oligotrophic ecosystems (i.e. freshwater and marine), where they tend to predominate due to having an advantage on photoautotrophic phytoplankton by obtaining limiting nutrients (e.g. P) through phagotrophy^31^,^32^,^33^,^34^. In addition to their flexible nutrition, mixotrophs seem also to be favoured by high light conditions^28^,^35^, and hence it would be expected that, under stabler and shallower upper mixed layers (UMLs) due to global change situation, they would be strongly benefited with respect to photoautotrophic groups by greater exposure to radiation.

Solar radiation including the UVR region of the spectrum, is another key factor that controls ecosystem functioning and their communities by acting upon several biotic targets (e.g. DNA, photosystems or membranes) and metabolic processes in planktonic organisms (e.g. photosynthesis, respiration, growth)^36^,^37^. Thus, the biological effects of UVR may be particularly striking in clear environments (e.g. open ocean areas and high-mountain lakes)^8^,^38^ because the high transparency of the water column allows the ambient UVR and photosynthetically active radiation (PAR) intensities to be sufficient to cause near-surface photoinhibition^39^.

Despite the ecological relevance of each of these environmental factors, the community’s responses to a single factor are frequently altered by synergistic or antagonistic interactions among them^40^. For example, some authors have reported that nutrient pulses can unmask the harmful UVR effects on phytoplankton and/or bacterial production (BP)^41^,^42^,^43^, altering the trophic interactions within the microbial loop^22^. Contrarily, other studies contend that nutrient pulses can attenuate the damage that UVR inflicts on PP and BP^44^,^28^,^45^.

However, no available study to date has experimentally quantified how the interaction between an increased nutrient-pulse frequency and high UVR levels will affect commensalistic phytoplankton–bacteria interactions, community dynamics (e.g. dominance and/or succession in the microbial community) and the ecosystem productivity in the future global-change scenario. To address these questions, we conducted a field mesocom experiment in lake La Caldera (Spain; Fig. S1) where we manipulated (1) the schedule of nutrient inputs (*press* [5 μg P L^−1^ × 6 times] vs. *pulse* [30 μg P L^−1^ once]), while maintaining the total amount of nutrients (i.e. P) at the end of the experiment, and (2) the spectrum of radiation received (with and without UVR). The rationale behind this approach was to mimic future increases in the frequency of atmospheric dust depositions rich in P from the Sahara Desert and high solar radiation levels (UVR and/or PAR) undergone by planktonic communities as if they were trapped in the top layer of the water column due to a potential stratification induced by global warming. We examine the structural, stoichiometric, and metabolic responses of phytoplankton and heterotrophic bacteria as well as the interaction between these two groups. In addition, we compare these experimental responses together with the microbial community dynamics and the natural variability in the atmospheric dust-deposition inputs entering the ecosystem via a 6-year seasonal and interannual observational study.

## Results

### Long-term biological and climatic data

**Figure 1:**
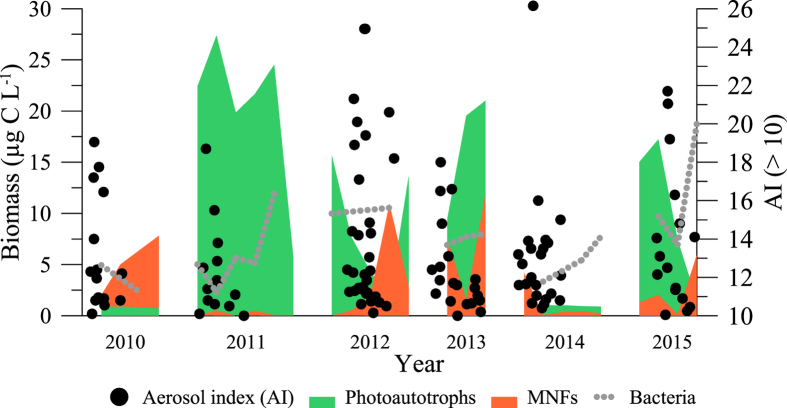
Dynamics of the biomass contribution (in μg C L^−1^) of main phytoplankton groups (mixotrophic nanoflagellates [MNFs, orange area] and photoautotrophs [green area]) and bacterial community [gray dotted line]) and aerosol index (AI in relative units, solid circles) with events >10 for the 2010–2015 ice-free period (from 1 June to 15 November) in lake La Caldera. Note that AI > 10 is a measure of intense atmospheric dust deposition inputs and that frequency is denoted with the degree of clustering of solid circles.

The temporal dynamics from 2010–2015 showed that phytoplankton predominated over bacteria in terms of biomass (except for 2015) and reflected two clear response patterns related to the intensity and frequency of aerosol inputs (i.e. Aerosol Index [AI]). Thus, when aerosol inputs were intense (AI > 10), as in the case of years 2011, 2013, and 2015, the biomass of photoautotrophs (mainly *Monoraphidium* sp.) considerably increased, whereas the biomass of MNFs (dominated by *Chromulina nevadensis*) remained low and unaltered (<5μg C L^−1^). By contrast, when aerosol inputs increased in frequency during 2010 and 2012 (i.e. the clusters of black dots in Fig. 1) the opposite pattern resulted, as reflected by the dominance of MNFs over photoautotrophs in La Caldera lake (Fig. 1). Ciliates and heterotrophic nanoflagellates (HNFs) were not detected on any sampling date during the 6-year period studied.

### Experimental approach

#### The lake’s physico-chemical conditions

The water temperature in the water column showed no thermal stratification (Fig. S2; Supplementary text S1). According to the attenuation coefficients of solar radiation (*k*_*d*305_ = 0.61; *k*_*d*320_ = 0.52; *k*_*d*380_ = 0.34), the first two meters received ca. 90% of incident UVR. Similarly, PAR also penetrated deep into the lake (*k*_*d*PAR_ = 0.25) with >10% of incident radiation reaching the bottom of the lake. Mesocosms in the experiments, incubated at 1-m depth, received mean irradiances 2- to 3-fold higher than those received by organisms in the water column (e.g. 11.04 vs. 4.06 [320 nm] μW cm^−2^ nm^−1^ and 908.60 vs. 468.86 [PAR] μmol photons m^−2^ s^−1^). Dissolved nutrients (total dissolved phosphorus [TDP] and total dissolved nitrogen [TDN], Supplementary Table S1) slowly decreased over time to non-detectable levels in the lake whereas dissolved organic carbon (DOC) remained relatively unchanged (Table S2).

#### Nutrient and chlorophyll a (Chl a) dynamics in mesocosms

In ambient nutrient treatments, we found a slight but significant rise in TDP (~0.16 μM P) at mid-term, followed by a significant fall at the end of the experimental period (Tables S1 and S3). In nutrient-enriched treatments, two well-differentiated patterns were discerned, depending on how nutrients were amended: (1) in *press* treatments, TDP increased up to 0.30 (±0.04) μM P under UVR at the end of the experimental period; and (2) in *pulse* treatments, TDP decreased after the nutrient pulse was added from ~0.40 (±0.05) μM P to concentrations similar to those registered before nutrient additions (31 August; Table S1). TDN remained relatively unchanged over the experimental period under ambient and *press* treatments (except for PAR_*press*_), whereas it was significantly higher under *pulse* treatments (Tables S1 and S3). Therefore, these results suggest that N was in excess and was not a limiting factor. DOC concentrations were low over the experimental period, particularly under ambient nutrient treatments (Tables S2 and S3); however, a continuous increase occurred under PAR_*pulse*_ treatment during the experiment. An interactive UVR × P × Time effect on TDP, TDN, and DOC was found (Table S3).

Similarly, there was a UVR × P × Time interactive effect on Chl *a* (Tables S4 and S5). By contrast, as a single factor, UVR did not exert a significant effect under ambient or enriched nutrient treatments (except for the final incubation day), whereas the addition of nutrients prompted a significantly steady increase in Chl *a* compared to ambient treatments.

#### Nutrient-pulse frequency and UVR effects on the microbial community

**Figure 2:**
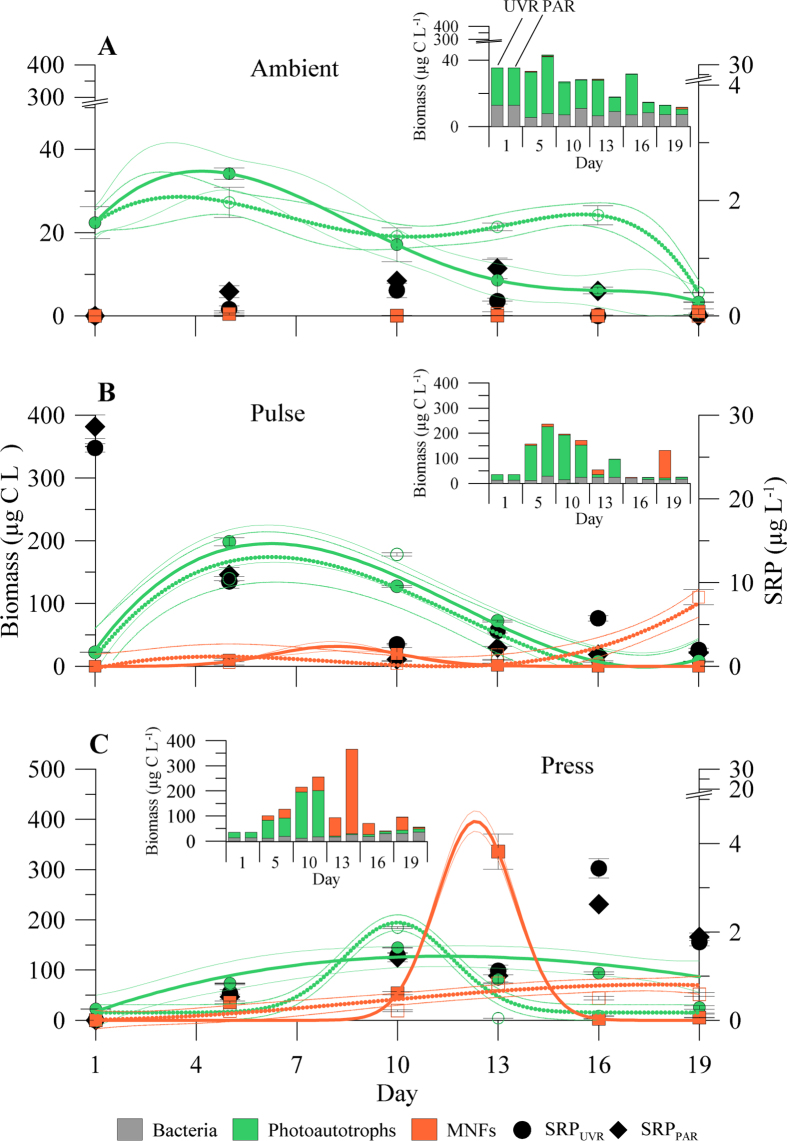
Non-linear regression of the dynamics of the main phytoplankton groups (in μg C L^−1^), mixotrophic nanoflagellates (MNFs, orange lines) and photoautotrophs (green lines) during the experiment under (**A**) ambient, (**B**) *press* and (**C**) *pulse* treatments. Dashed and solid thick lines (green and orange) represent samples under UVR (>280 nm) and PAR (>400 nm), respectively, whereas thin dashed and solid lines (green and orange) indicate 95% interval confidence for each regression fitted by peak-Gaussian: y = y_0_ + a exp (−0.5 ((x − b)/w^2^)) or cubic: y = ax^3^ + bx^2^ + cx + d. Solid diamonds and circles in A,B and C panels represent the temporal variation of soluble reactive phosphorus (SRP, in μg L^−1^) over the experiments under two radiation treatments: UVR (>280 nm) and PAR (>400 nm), respectively. Inserted figures in (**A**,**B** and **C**) represent total community biomass including bacterial compartment (in μg C L^−1^; gray) under the two radiation treatments mentioned previously.

Phytoplankton dominated the biomass of the nanoplanktonic food web both in ambient and nutrient-amended treatments over the experiment (Fig. 2A–C; Tables S5 and S6). However, as for the lake, ciliates and HNFs were not found in any of the mesocosms. Bacterial biomass remained relatively stable (values <13 μg C L^−1^) over the experiment regardless of the nutrient and radiation treatments considered (insert in Fig. 2B,C; Tables S5 and S6).

Two phytoplankton species dominated the community under all experimental conditions (see below), *Monoraphidium* sp. (a photoautotroph) and *Chromulina nevadensis* (a MNF), which comprised over 95% of the total phytoplankton biomass during the experiment. The rest of the total biomass was represented by Bacillariophyeae (i.e. *Cyclotella* sp.), Dinophyceae (i.e. *Gymnodinium* sp.), and Cryptophyceae (i.e. *Rhodomonas* and *Cryptopmonas* sp.). At the beginning of experiment, no significant differences (*F* = 2.25, p = 0.19) were found in the total phytoplankton (photoautotrophs and MNFs) biomass between the ambient (20.40 ± 3.82 μg C L^−1^) and nutrient-enriched treatments (*press* = 26.79 ± 4.83 and *pulse* = 19.96 ± 4.52 μg C L^−1^). However, over the experiment, the photoautotrophs and MNFs biomass decreased in ambient treatments whereas they increased in the *press* and *pulse* treatments. Thus, photoautotrophs dominated the microbial community in the ambient nutrient treatments, contributing between ca. 6 to ca. 28 μg C L^−1^, while MNFs biomass did not exceed 1 μg C L^−1^ (Fig. 2A). In both nutrient treatments (*press* and *pulse*) photoautotrophs were replaced by MNFs, although with different timing, as they dominated on day 13 in the case of *press* treatments and on day 16 for *pulse* treatments. In fact, in the *pulse* treatments, independently of the radiation treatment, photoautotrophs grew exponentially up to day 7 (ca. 200 μg C L^−1^), after which they progressively declined towards the end of the experiment. By contrast, MNFs followed the opposite pattern, from an initially low biomass (<20 μg C L^−1^) to a maximum of ca. 110 μg C L^−1^ at the end of the experiment under UVR. In the case of the *press* treatment under UVR, photoautotrophs showed a unimodal response and reached their maximum biomass by day 10. The subsequent decline in biomass after this date was coupled with a sharp increase in MNFs towards the end of the experiment. A similar unimodal response pattern was observed for MNFs under PAR-treatment, although biomass peaked by day 13 (ca. 400 μg C L^−1^), whereas the photoautotrophs biomass remained fairly constant throughout the experiment (Fig. 2C; Tables S5 and S6).

#### Nutrient-pulse frequency and UVR effects on metabolic variables

**Figure 3:**
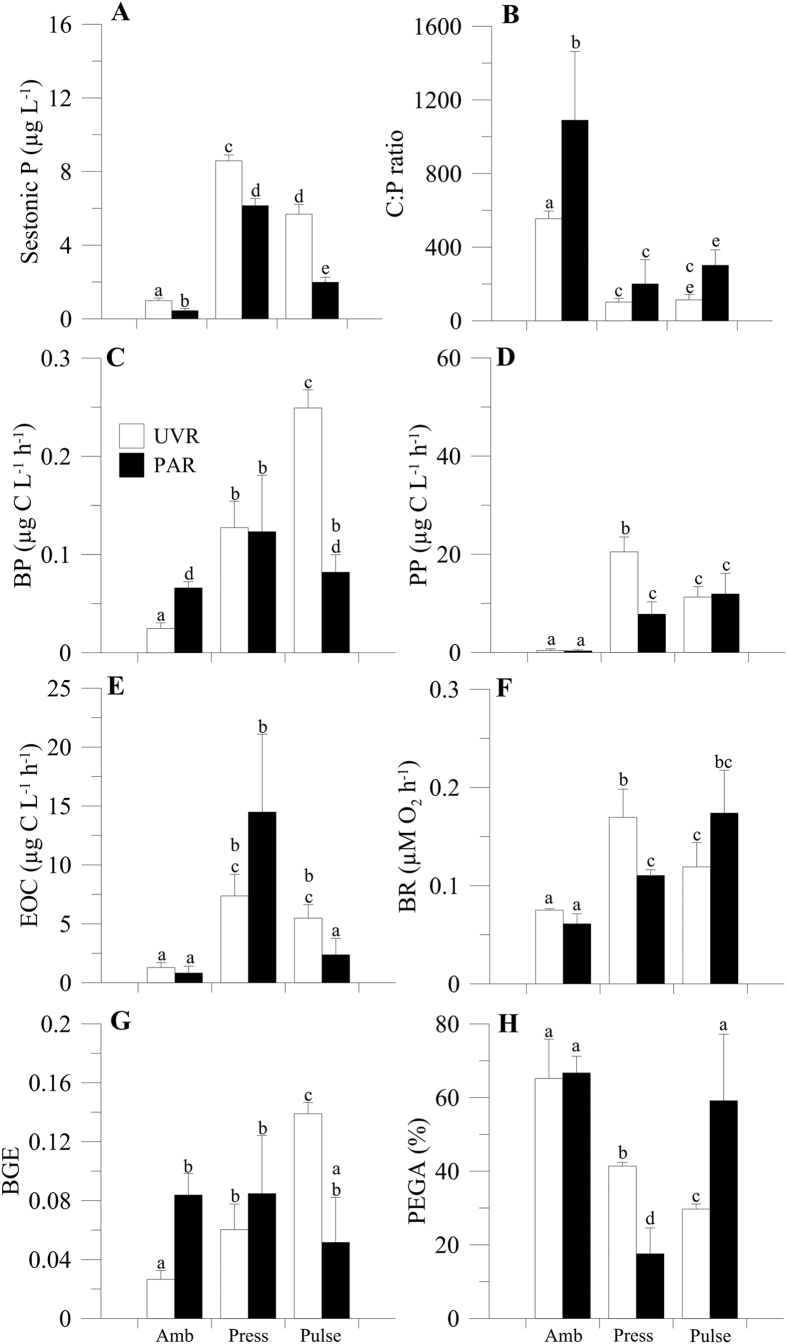
(**A**) Sestonic phosphorus (P) concentrations (in μg L^−1^), (**B**) sestonic carbon: phosphorus ratio (C:P ratio), (**C**) bacterial production (BP, in μg C L^−1^ h^−1^), (**D**) primary production (PP, in μg C L^−1^ h^−1^), (**E**) excretion of organic carbon (EOC, in μg C L^−1^ h^−1^), (**F**) bacterial respiration (BR, μM O_2_ h^−1^), (**G**) bacterial growth efficiency (BGE) and (**H**) percentage of photosynthetic excreted gross assimilation (%PEGA) in lake La Caldera under two radiation treatments: UVR (>280 nm, white bars) and PAR (>400 nm, black bars) and three nutrient treatments: ambient (amb), *press* and *pulse*. The bars represent mean values of three replicates and lines in top of the bars are the standard deviation. Letters indicate differences among treatments by Bonferroni *post hoc* test. Note that these results represent responses observed over the longer term, when communities received the same amount of nutrients but at different frequencies.

At the end of experiment, when samples had received the same amount of nutrients but at different frequencies, the percentage of photosynthetic excreted carbon gross assimilation (%PEGA) exhibited values <100% in all cases (Fig. 3H), as did the ratio bacterial carbon demand:excreted organic carbon (BCD:EOC ratio; Fig. S3A), and showed the same pattern of response to UVR and nutrient treatments as the BCD:EOC ratio (Fig. S3A; Table S7). In addition, values <100% for PEGA and the BCD:EOC ratio were also found in the shorter term, when both communities had received a different intensity of nutrient pulses (moderate and intense treatments; Fig. S3B; Tables S7 and S8 and Supplementary text S2).

Sestonic P, PP, bacterial respiration (BR), and %PEGA (Fig. 3; Table S9) were significantly higher under the *press* treatment when compared to the *pulse* treatment and UVR; however, the opposite was true for BP and the bacterial growth efficiency (BGE), where the lowest values corresponded to the *press* treatment. By contrast, under UVR, the sestonic C:P ratio and EOC were similar between the *press* and *pulse* treatments. In this sense, and partially contrasting with the findings over the shorter term (moderate and intense treatments; Fig. S4; Table S8 and Supplementary text S2), our results show that the *press* or *pulse* input counteracted (e.g. C:P ratio, BP, BGE) or reversed (e.g. sestonic P, PP, BR, %PEGA) the negative UVR effects, except for the %PEGA, for which a *pulse* input unmasked the negative effects mentioned above.

#### Relationship between the microbial community and its environment

**Figure 4:**
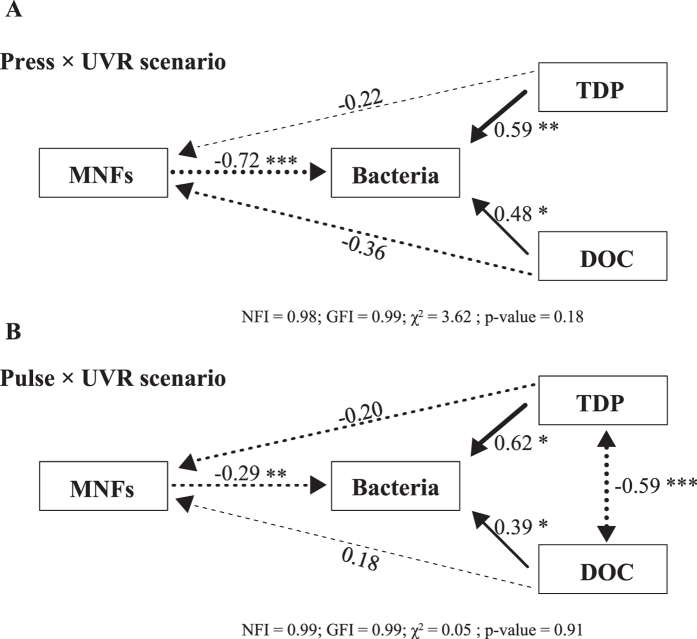
Graphic path model representing the relationship between mixotrophic nanoflagellates (MNFs) and bacterial biomass and the dynamics of nutrients (total dissolved phosphorus, TDP and dissolved organic carbon, DOC) in lake La Caldera under the two ‘natural’ scenarios of frequency of pulsed nutrients considered, (**A**) *press* × UVR and (**B**) *pulse* × UVR. Arrow widths are proportional to path coefficients; the two-headed arrow denotes correlations and the one-headed arrow causal relationship. Negative effects are indicated by dashed lines and positive effects by solid black lines. Numbers join to the paths indicate standardized paths coefficients. Significance p-values are denoted with *p-value < 0.05, **p-value < 0.01 and ***p-value < 0.001. Fit statistics (Goodness-of-fit index, GFI; normal-fit index, NGI; χ^2^; p-value).

Due to the variation in the interaction strength of the microbial community and the changes in the taxonomic composition throughout the incubation period, a structural equation modelling (SEM) analysis was performed to test the potential top-down (by MNFs) vs. bottom-up (by DOC and TDP) control on the bacterial biomass under the two simulated natural scenarios considered in this study (*press* × UVR and *pulse* × UVR). The two SEM analyses exhibited a good fit with the observed data for both scenarios, as indicated by their non-significant χ^2^ (p > 0.05) and by the two goodness-of-fit indices (NFI and GFI >0.9 in both cases; Fig. 4 and supplementary text S3). Regardless of the nutrient treatment, SEM yielded positive and significant (p < 0.05) standardized path coefficients for the effects of DOC and TDP on bacteria (*press*, 0.48 and 0.59; *pulse*, 0.39 and 0.62, respectively), whereas they were negative and non-significant (p > 0.05) for MNFs. In addition, both resources were positively correlated in the *pulse* × UVR scenario (p < 0.001) (Fig. 4B). SEM analysis showed negative and significant standardized path coefficients for conditions (i.e. low P) favouring MNFs, resulting in an increased MNFs bacterivory on the bacterial biomass, which was 2.5-fold higher in the *press* compared to the *pulse* × UVR scenario (*press*, −0.72 and p < 0.001; *pulse*, −0.29 and p < 0.01).

## Discussion

This study responds to the increasing need for forecasts on how the growing frequency of climatically driven nutrient pulses and their interaction with other stressors alters the structure and the strength of biotic interactions in food webs^2^. Our findings show that under UVR the *press* conditions, more than *pulse* conditions, stimulated PP and phytoplankton biomass. Although previous studies also reported a concomitant microbial loop development after nutrient inputs in oligotrophic ecosystems^17^,^46^, our results show that this may not generally be the case for all oligotrophic ecosystems, supporting previous studies in this ecosystem that found only a transitory development of this compartment after nutrient pulses^21^,^47^.

Surprisingly, the limited bacterial development and the absence of ciliates and HNFs was counterbalanced by the increased development of MNFs, particularly under UVR and *press* conditions. In fact, under *pulse* conditions, photoautotrophs dominated the community exhibiting a characteristic domed-curve dynamic probably due to the rapid and high levels of nutrient availability that caused an overshoot of the consumer-carrying capacity, generating rapid community growth followed by substantial declines, which in some cases, even lead to population extinctions^48^. By contrast, the early and sustained development of MNFs under the *press* conditions (particularly under UVR) is consistent with previous findings, both in laboratory and field studies^30^,^33^,^49^,^50^, showing that MNFs are most successful when nutrients are scarce and solar radiation plentiful. This is indicative that the *press* conditions maintained nutrient scarceness (as actual availability) below the threshold that would elicit a bloom-development of photoautotrophic phytoplankton.

Our field survey on a seasonal and inter-annual scale and our experimental results show a consistent response pattern with the prevalence of MNFs under low but frequent nutrient inputs, whereas photoautotrophs responded to high nutrient inputs, denoting the differential growth strategy that both groups adopted with the nutrient-input schedule. However, in neither approach was there a clear development of bacteria nor was the presence of ciliates or HNFs detected, a finding that we attribute to a higher competitive advantage of MNFs over ciliates and HNFs (not found here), because MNFs have a lower minimum threshold of bacteria abundance to grow^51^. In addition, as Mitra *et al*.^30^ recently suggested, the synergistic co-operation between phototrophy (to obtain C) and phagotrophy (to obtain nutrients) within the same organism also confer higher competitive advantage to MNFs over photoautotrophs. This is particularly relevant under high light intensities and low nutrient concentrations, conditions which favour increased bacterivory rates^31^ and positive net growth rates^50^ of MNFs.

In relation to the joint effects of pulsed nutrients and UVR on primary producers, bacteria and their commensalistic interaction, our results show that PP and BP were stimulated several-fold over the shorter term (different intensity of nutrient inputs; Fig. S4 and Supplementary text S2) and over the longer term of the experiment (equal intensity of nutrient inputs; Fig. 3). The reported increases in PP were consistently coupled with increases in the sestonic P content and decreases in the sestonic C:P ratio, indicating a more balanced elemental content of phytoplankton to growth^43^.

Despite the EOC satisfying the BCD in both nutrient-input schedules, the BCD (and BR) was higher (2-fold) and the BP (and BGE) was lower in the *press* × UVR than in the *pulse* × UVR scenario (Fig. 3C). These findings suggest that *press* conditions caused bacteria to use most of their C uptake for energy expenditure rather than for biomass production. Moreover, this lower BP found under a *press* than *pulse* × UVR scenario could be due to a higher bacterivory by MNFs on bacteria, a possibility supported by the fact that the negative SEM coefficients for the MNF–bacteria interaction were 2-fold higher under the *press* × UVR scenario, hence denoting an intensive top-down control of bacterial compartment by MNFs. These results support our previous findings on the dominance of MNFs in oligotrophic ecosystems, and are in line with a newly proposed microbial food-web paradigm^31^,^30^ where MNFs dominate the microbial community and are responsible for most C fixation, while also controlling the bacterial biomass due to their phagotrophic metabolism. Therefore, the energetic constraint of bacteria (i.e. a higher respiratory cost) together with a higher top-down control under *press* conditions could help to explain the steadiness of the heterotrophic compartment over the time.

From the above, we conclude that the growing frequency of pulsed nutrients entering into the ecosystems together with high UVR fluxes can reinforce the dual control that MNFs exert on bacteria via increased photosynthetic C release, a share of which can be recycled through phagotrophy following the MNF–bacteria–MNF sequence. This dual form of control favours the bypass or short circuit between the two functional groups, as described by Medina-Sánchez *et al*.^31^ and Carrillo *et al*.^52^ in freshwater ecosystems and by Ptacnik *et al*.^35^ in marine ecosystems. This mutualistic interaction underpins the mixotrophic strategy reported in this study, as a high frequency of low nutrient inputs under high UVR levels benefits the growth of MNFs. Therefore, the combination of these two factors could explain the inter-annual persistence of MNFs in this and other freshwater and marine clear-water ecosystems^31^,^33^,^53^.

The present study has three major implications. First, current changes that are occurring in resources (e.g. the increasing frequency of pulsed nutrients) and energy fluxes (i.e. higher solar radiation under shallower UMLs) in many oligotrophic areas worldwide may cause a shift in the dominance of the planktonic community from photoautototrophs to mixotrophs coupled with higher primary productivity rates in these ecosystems. Second, such changes in planktonic community structure could reinforce the top-down control on heterotrophic bacteria through mixotrophic metabolism. Third, it is critical to consider the interaction between chronic and stochastic environmental stressors to more accurately predict and understand how population and communities will respond to the predicted climate-driven changes.

## Methods

### Study site

La Caldera is a high-mountain lake situated above the treeline (3050 m a.s.l.) in the Sierra Nevada National Park (southern Spain, 36°55′–37°15′N, 2°31′–3°40′W) on siliceous bedrock in a glacial cirque. The site has been previously described in terms of its physical, chemical, and biological characteristics, highlighting its high transparency to solar radiation, strong limitation by P and relatively simple pelagic community^47^.

### Measurements of natural inputs of pulsed nutrients: Remote sensing

As a measure of aerosol content in the troposphere, we used the ultraviolet (UV)-AI produced by the Giovanni online data system, developed and maintained by the NASA GES DISC^54^ (Supplementary text S4) as a proxy for natural nutrient inputs into the ecosystem.

### Observational approach: Field sampling in the water column from 2010–2015

Sampling was conducted during the ice-free periods (1 June to 15 November) from 2010 to 2015. For each sampling day, a lake sample was constructed from equal volumes of lake-water samples collected with a 6-L horizontal Van Dorn sampler from three depths, spaced evenly within the photic layer at least affected by 1% PAR. From this composite lake sample, subsamples of 125 mL for phytoplankton taxonomic composition and 500 mL for ciliates and HNFs were preserved using alkaline Lugol’s reagent.

### Experimental approach

An *in situ* experimental setup consisted of 18 UVR-transparent low-density polyethylene mesocosms (0.58 m in diameter, LDPE, Plasticos Andalucia), closed at the bottom and the top, with a total volume of 0.1 m^3^. The LDPE used transmits >70% of UVR and >85% of PAR. A water pump was used to fill each mesocosm with 45 μm of filtered lake water (excluding zooplankton), which was collected from the first meter in depth. Once filled, the mesocosms were set in six racks 4 m long × 3 m wide (three for UVR and three for PAR, see below) made of 3-cm polyvinyl chloride pipe, and placed under a thin water layer in the lake (1 m deep). Thus, the cells were exposed to the worst-case scenario of solar radiation (i.e. as if they were in the top layer of the water column due to a shallower UML), as is expected due to global warming^55^,^56^. The six racks were set approximately 25 m apart to avoid shading effects and were secured to a buoy attached to an anchored rope. The radiation treatments were performed by using a cover of polyethylene arranged on the mesocosms that transmitted >70% of UVR and >85% of PAR in the case of the UVR treatment, whereas the PAR treatment was performed by using a cover of UV-filter foil (UV-Process Supply Inc., IL, USA) arranged on the mesocosms that transmitted >90% of PAR but blocked UVR (<400 nm).

The experiment was conducted from 31 August to 18 September 2011 using a 2 × 3 full-factorial matrix (in triplicate) with: a) two radiation treatments, UVR (>280 nm) and PAR (>400 nm); and b) three nutrient conditions: (1) ambient (amb); (2) *press* (5 μg P L^−1^ × 6 times); and (3) *pulse* (30 μg P L^−1^ × 1 time). When we integrated over the experimentation period, both the *press* and *pulse* treatments had the same quantity of nutrients added. To ensure that P (as NaH_2_PO_4_) remained as the limiting nutrient in the enclosures, inorganic N (as NH_4_NO_3_) was added to mesocosms to reach an N:P molar ratio of 30. The rationale for using nutrient additions (N and P) as a proxy of Saharan dust deposition was because: (1) mineral aerosols are the dominant source of atmospheric TP on a global scale (82%)^6^ and (2) previous studies in the Sierra Nevada National Park area established clear connections between TP loads and Saharan dust depositions^57^. Also, the addition of inorganic N to reach an N:P molar ratio of 30 mimics the mean value of the molar TN:TP ratio also found in total atmospheric deposition^57^.

At the beginning of the experiment, nutrients were added for both treatments (5 μg P L^−1^ for the *press* treatment and 30 μg P L^−1^ for the *pulse* treatment), and on days 4, 7, 9, 12, and 15 of September in the case of the *press* treatment until a final concentration of 30 μg P L^−1^ was reached. The duration of the experiment was based upon the regional frequency of atmospheric deposition events that are involved with the release of P into lake La Caldera^57^ and with the microbial community dynamics (Supplementary text S5). Samples for the different analysis and measurements outlined below were taken early in the morning (and before the nutrient additions in the case of the *press* treatment) in each experimental day using a water pump connected to a silicone tube inserted into each mesocosm during the incubation period to prevent tampering.

### Analyses and measurements

#### Stoichiometric variables

To determine sestonic C and P, we filtered water samples from each mesocosm (500 mL) through pre-combusted (1 h at 550 °C) GF/B Whatman filters (25-mm diameter). For sestonic P, filters were placed in acid-washed vessels, persulfate digested at 120 °C for 30 min and immediately analysed as SRP following Murphy & Riley^58^. For sestonic C, filters were desiccated (24 h at 60 °C) and analysed using an elemental analyser (Perkin Elmer 2400, USA).

#### Structural variables

Water samples (250 mL) for Chl *a* determination were filtered through GF/F Whatman filters (25-mm diameter) and frozen at −20 °C until analysis. For the Chl *a* analyses, filters were placed in centrifuge tubes (15 mL) with 5 ml of 90% acetone for 24 h at 4 °C in darkness. After this, the samples were centrifuged and the supernatant fluorescence was measured with a fluorometer (Perkin Elmer LS 55, USA)^59^.

The abundance of phytoplankton (photoautotrophs and MNFs), HNFs and ciliates were quantified following the procedure described by Straskrabová *et al*.^60^. Bacterial abundance was determined by the flow-cytometry technique (FACSCanto II, Becton Dickinson Biosciences, Oxford, UK). Previously, 1.5 mL of sampling water was fixed with 75 μL of particle-free 20% (w/v) paraformaldehyde (1% final concentration) and frozen in liquid nitrogen to be stored at −80 °C until analysed^61^,^62^ (Supplementary text S6).

#### Functional variables

PP was measured with the ^14^C-incorporation method^63^. Briefly, sets of four 50-mL quartz vessels (three clear and one dark) by treatment, with 0.37 MBq of NaH_14_CO_3_ (DHI Water and Environment, Germany) added, were placed *in situ* at 1 m under the surface, receiving a radiation treatment identical to that of the mesocosms, and incubated for 4 h symmetrically distributed around noon. Total organic carbon (TOC) was measured in 4-mL subsamples collected before filtration. Particulate PP was determined by serial filtration of the entire content of each quartz vessel (50 mL) through 1-μm (particulate organic carbon, POC_1_) and 0.2-μm (particulate organic carbon, POC_2_) pore-size Nucleopore filters of 25 mm in diameter. EOC was calculated as the sum of DOC and POC_2_ (Supplementary text S7).

BP was measured using the radio-labelled thymidine-incorporation technique^64^. A set of five (3 + 2 blanks) acid-cleaned and sterilised tubes per treatment containing 1.5 mL of sample were inoculated with ^3^H-thymidine (SA = 48–50 Ci mmol^−1^, Perkin Elmer) to a final saturating concentration of 12 nM. After this, tubes were incubated at the *in situ* temperature for 1 h in darkness (Supplementary text S7).

Samples for BR (<0.7-μm fraction) were filtered through Whatman GF/F filters (25-mm diameter), placed in sealed 25-mL glass vessels equipped with optode sensor-spots (SP-PSt3-NAU-D5-YOP) and incubated in darkness in a temperature-controlled bath to maintain the same temperature as the lake and to measure the oxygen concentration over time using an oxygen optode (Fibox 3, PreSens GmbH, Germany; Supplementary text S7).

BGE was estimated as BP divided by the sum of BP and BR^65^,^66^. As autochthonous C (measured as EOC) is the C source preferentially used by bacteria^22^,^67^,^68^, and given the feasibility of segregating microbial fractions through filtration, we estimated the PEGA as:


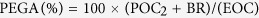


This represents a direct measurement of the strength of the interaction between phytoplankton and bacteria.

#### Data and statistical analysis

The effect of nutrients (ambient, *press* and *pulse*) on initial C biomass was tested by one-way analysis of variance (ANOVA). The interactive effect of UVR and P on phytoplankton and bacteria biomass and Chl *a* over time was tested by two-way repeated measures ANOVA (RM-ANOVA). The interactive effects of UVR and P evaluated as the intensity (moderate vs. intense) and frequency (*press* vs. *pulse*) of the pulse on stoichiometric (sestonic P and sestonic C:P ratio) and functional variables (BP, PP, EOC, BR, BGE, and %PEGA) were tested by a two-way ANOVA. Shorter- and longer-term responses were analysed separately because of the different nutrient concentrations present in the mesocosms each day. Sphericity (by Mauchly’s test), homoscedasticity (by Cochran’s and Levene’s tests) and normality (by Shapiro-Wilk’s test) were checked for each variable to verify the ANOVA and RM-ANOVA assumptions, respectively. When interactive effects were significant, Bonferroni’s *post hoc* test was used to denote statistical differences among and within treatments.

SEM analysis was used to test whether the pool of the main resources (i.e., TDP and DOC) influenced the relationship between MNF and bacteria biomass at the end of the experimental period under the two ‘natural’ scenarios considered (i.e., *press* × UVR and *pulse* × UVR, Supplementary text S3).

## Additional Information

**How to cite this article:** Cabrerizo, M. J. *et al*. Rising nutrient-pulse frequency and high UVR strengthen microbial interactions. *Sci. Rep.* **7**, 43615; doi: 10.1038/srep43615 (2017).

**Publisher's note:** Springer Nature remains neutral with regard to jurisdictional claims in published maps and institutional affiliations.

## Supplementary Material

Supplementary Information
