# Supplementary material for: Rising nutrient-pulse frequency and high UVR strengthen microbial interactions
Source: Sci Rep. 2017 Mar 2;7:43615. doi: 10.1038/srep43615 (PMC5333626; doi:10.1038/srep43615)
Supplement: Supplementary Information [file srep43615-s1.pdf]

Supporting information for

Rising nutrient-pulse frequency and high UVR strengthen microbial interactions

Marco J. Cabrerizo[1, 2, *], Juan Manuel Medina-Sánchez[1], Irene Dorado-García[2], Manuel Villar-Argaiz[1], Presentación Carrillo[2]

[1] Departamento de Ecología, Facultad de Ciencias, Universidad de Granada, Campus Fuentenueva s/n, 18071, Granada (España)

[2] Instituto Universitario de Investigación del Agua, Universidad de Granada, C/ Ramón y Cajal, 4, 18071, Granada (España)

*Corresponding author: Marco J. Cabrerizo, mjc@ugr.es

Supplementary text S1

Measurements of physical-chemical variables: Vertical profiles of solar radiation in the water column were measured at noon during the first experimental day using a submersible BIC Compact 4-Channel radiometer (Biospherical Instruments Inc., CA, USA) with three channels in the UVR region (305, 320, and 380 nm) and one broad-band channel for PAR (400-700 nm). Diffuse attenuation coefficients for downward irradiance (k_d) were determined from the slope of the linear regression of the natural logarithm of downwelling irradiance *vs.* depth for each wavelength range considered ($n > 300$, $R^2 > 0.90$). Temperature profiles were recorded using a multiparametric probe (Hanna HI9828-0).

Water samples for the chemical determination of total dissolved phosphorus (TDP), soluble reactive phosphorus (SRP), and total dissolved nitrogen (TDN) were also collected, and prior to determination were filtered through GF/F Whatman filters (25-mm diameter). Samples for TDP were persulfate digested at 120ºC for 30 min and determined (as for SRP) using 10 cm quartz cuvettes, following the acid molybdate technique [1]. TDN samples were also persulphate digested and measured as NO_3^- following the ultraviolet spectrophotometric method [1]. Water samples for dissolved organic carbon (DOC) determination were filtered through pre-combusted (2 h at 500ºC) GF/F Whatman filters (25-mm diameter); after this, they were acidified with 1N HCl (2%) and stored in darkness at 4ºC until analysis. DOC concentrations were measured with the high-temperature catalytic oxidation method in a TOC analyzer (Shimadzu, model 5000) [2].

Supplementary text S2

Nutrients pulse intensity and UVR effects on metabolic variables: The percentage of photosynthetic excreted gross assimilation (%PEGA), from a direct measurement of carbon (C) incorporated by bacteria of photosynthetic origin, was consistently < 100% in all treatments, and

showed a similar pattern to that of the bacterial carbon demand: excreted organic carbon (BCD: EOC) ratio (Fig. S3A; Table S7), suggesting that the C released by phytoplankton was the main C source supporting BCD.

In general, the addition of a moderate or intense pulse increased sestonic P and all metabolic variables, but decreased sestonic carbon: phosphorus (C:P) ratio with respect to ambient conditions. However, a combined impact of UVR and a moderate pulse counteracted (e.g., EOC, BGE) or reversed (e.g., BP, BR) the negative UVR effects, whereas an intense nutrient pulse unmasked (e.g., sestonic P, PP, BR, %PEGA) or even enhanced (e.g., BP, EOC) these negative UVR effects (Fig. S4; Table S8).

Supplementary text S3

Structural equation modeling (SEM): We performed a SEM analysis to test the top-down (mixotrophic nanoflagellates, MNFs) vs. bottom-up control (DOC and TDP) on bacteria biomass under the two scenarios considered in this study (UVR × *press* and UVR × *pulse*), due to the variation in the interaction strength of the microbial community and the changes in the taxonomic composition through incubation. As the sample size was relatively small (18 data per variable), we followed the recommendations of Tanaka et al. [3]. Previously, all variables considered were assessed for normality before statistical analysis. Suitable transformation (log) was performed for bacterial biomass to improve normality according to Zar [4]. The GLS→ML method with 300 iterations was used to estimate standardized path coefficients in our models, and the χ^2 to test the degree of fit of the model to the observed data. Non-significant χ^2 indicates that the pattern predicted by the hypothesis is not different from the observed data, and thus the model can be accepted [5]. Notwithstanding, it is generally accepted that the χ^2 test should be

interpreted with caution and supplemented with other goodness-of-fit indices [6]. Therefore, two additional other goodness-of-fit indices were used, such as the Bentler-Bonnet Normed Fit Index (NFI) and the Goodness-of-Fit Index (GFI).

Supplementary text S4

Measurements of natural inputs of pulsed nutrients: Remote sensing: Aerosol data were successfully used for the study of Saharan dust inputs in previous studies of this freshwater ecosystem, due to the highly positive correlation of TOMS aerosol index (AI) with total phosphorus (TP) [7, 8]. We used TOMS AI data for the 2010-2015 ice-free period at lake La Caldera (37.5ºN, 3.5ºW). We considered that values of AI > 10 represent intense deposition events. Original data for this study were downloaded from Giovanni database web [9].

Supplementary text S5

Experimental approach: The integrated approach used here is sufficiently realistic to unravel the underlying mechanisms that govern the interaction between nutrient pulses, UVR, and the microbial community in the ecosystem because (i) the incubation period extended over three weeks, allowing us quantify the microbial loop response and changes in phytoplankton community; (ii) the UVR and nutrients pulse applied reproduced the natural optical conditions in the water column and the intensity and frequency of atmospheric deposition of aerosol in this area [7, 8]; and (iii) the absence of zooplankton in the mesocosms did not alter the response pattern of the microbial community structure with respect to natural lake conditions, suggesting a negligible net effect of zooplankton on the response of the microbial dynamics.

Supplementary text S6

Phytoplankton abundances: An aliquot of 50 mL from each sample was settled in an Utermöhl chamber of 2.6 cm diameter for 48 h to ensure complete sedimentation of the smallest phytoplankton species and counted at 400× and 1000× magnification under an inverted microscope (Carl Zeiss AX10, LCC, USA). For each sample, at least 400 cells of the more abundant phytoplanktonic species were counted, and 20 cells per species were measured for each date to estimate cell volume according to a corresponding geometrical shape. Phytoplankton biomass was estimated by approximating the cell volume to their geometric shape [10] and transform it to carbon (C) units following Rocha & Duncan [11]. For ciliates and heterotrophic nanoflagellates (HNF), an aliquot of 300-mL of each sample was allowed to settle for 72 h, and the supernatant was removed by suction with a Pasteur pipette coupled to a low-pressure pump. The remaining 50 mL were again settled in an Utermöhl chamber with the same procedure as described above for phytoplankton counting.

Bacteria abundances: Before analyses, the samples were unfrozen and stained with Sybr®Green I DNA (Sigma-Aldrich) 1:5000 final dilution of initial stock [12, 13]. A standard concentration of yellow-green 1-μm beads (10^5 particles mL^{-1}) (Fluoresbrite Microparticles, Polysciences, Warrington, PA, USA) was added in order to determine absolute cell concentrations [13, 14]. Bacterial biomass was estimated by approximating cell volume to their geometric shape and to transform it to C units following suitable conversion factors [15].

Supplementary text S7

Primary production (PP): All filtrations were done under low vacuum pressure (< 100 mm Hg) to minimize cell breakage. All the filters were placed in scintillation vials and also

acidified with 1 N HCl (2%) for 24 h to remove inorganic radiocarbon before the addition of liquid scintillation cocktail (Ecoscint A). After this, all samples were measured using a scintillation counter equipped with autocalibration (Beckman LS 6000 TA). The filtrate < 0.2 µm obtained was also treated as described for the total organic carbon (TOC, see main text). Because of the absence of photoautotrophic picoplankton [16] and not significant retention of bacteria in 1-µm pore-size filters, the organic ^{14}C retained on the 0.2-µm pore filters corresponded to the phytoplankton exudates incorporated by heterotrophic bacteria (POC_2) [17, 18].

Bacterial production (BP): Extraction was carried out with 5% (final concentration) cold trichloroacetic (TCA). Immediately, the tubes were centrifuged at 16000 g, rinsed twice with 5% TCA and measured in a scintillation counter equipped with autocalibration (Beckman LS 6000 TA). Data were corrected by blanks (bacteria were killed with 5% TCA before the addition of the radiotracer). We applied the conversion factors 1×10^{18} cells mol^{-1} of thymidine [19] and 2×10^{-14} g C per cell [20] to convert incorporated traces into BP expressed in C terms.

Bacterial respiration (BR) measurements: Oxygen-concentration measurements were made in darkness using an optic-fiber oxygen transmitter (Fibox 3; PreSens GmbH, Germany) with temperature compensation to register any change in the bath temperature, and connected to a computer with Oxyview 6.02 software to register the data. Prior to experimentation, the system was submitted to a two-point calibration, together with data of atmospheric pressure and temperature. Firstly, 0% O_2 concentration was determined by adding sodium sulfite (Na_2SO_3) to sterilized-distilled water to a final concentration > 0.1 mg mL^{-1}. To achieve the 100% O_2 saturation, wet cotton wool was put into the closed flask to ensure that the air was over-saturated with O_2 by water vapor. BR rates were calculated from least-square regressions after confirming that oxygen fitted a linear model during the first 24 h after sampling.

Bacterial carbon demand: The bacterial carbon demand (BCD) was calculated as the sum of BP plus BR.

Supplementary tables:

Table S1: Mean concentration (± SD) of total dissolved phosphorous (TDP) and nitrogen (TDN) (in µM) measured during the incubation period in each experimental treatment (radiation [UVR, > 280 nm; PAR, > 400 nm] and nutrients [ambient (amb), *press*, and *pulse*]) and in the water column of lake La Caldera. Data from 31 August represent concentrations before nutrient additions. n. d. represents non-detectable TDP or TDN concentrations.

				TDP			
Day	Lake	UVR$_{amb}$	PAR$_{amb}$	UVR$_{\mathit{press}}$	PAR$_{\mathit{press}}$	UVR$_{\mathit{pulse}}$	PAR$_{\mathit{pulse}}$
31 August				0.06 (0.01)			
4 September	0.06 (0.00)	0.03 (0.01)	0.04 (0.01)	0.03 (0.01)	0.08 (0.01)	0.41 (0.03)	0.39 (0.07)
9 September	0.02 (0.01)	0.15 (0.03)	0.14 (0.00)	0.25 (0.03)	0.12 (0.07)	0.27 (0.06)	0.11 (0.03)
12 September	0.01 (0.00)	0.16 (0.00)	0.01 (0.00)	0.16 (0.02)	0.09 (0.01)	0.23 (0.04)	0.08 (0.02)
15 September	0.01 (0.00)	0.10 (0.08)	0.01 (0.00)	0.23 (0.03)	0.08 (0.01)	0.16 (0.00)	0.06 (0.01)
18 September	n.d.	0.04 (0.00)	0.02 (0.00)	0.30 (0.04)	0.07 (0.01)	0.08 (0.03)	0.05 (0.01)
				TDN			
31 August				20.00 (1.43)			
4 September	17.86 (2.86)	12.86 (24.28)	1.43 (1.42)	24.30 (40.71)	n.d.	21.43 (41.43)	0.71 (0.00)
9 September	18.57 (0.07)	17.14 (24.29)	20.00 (20.43)	20.01 (32.14)	5.71 (2.14)	30.00 (35.71)	0.71 (0.02)
12 September	14.30 (0.01)	12.14 (25.00)	20.01 (40.79)	12.14 (36.43)	0.71 (5.00)	40.71 (32.86)	40.71 (1.43)
15 September	9.70 (3.57)	15.00 (32.14)	50.71 (25.50)	14.28 (32.85)	0.72 (4.29)	19.29 (32.85)	1.43 (2.86)
18 September	n.d.	20.00 (45.71)	50.72 (20.43)	30.00 (33.57)	21.43 (2.86)	47.86 (27.14)	16.43 (2.14)

Table S2: Mean concentration (± SD) of dissolved organic carbon (DOC, in μM) measured during the incubation period in each experimental treatment (radiation [UVR, > 280 nm; PAR, > 400 nm] and nutrients [ambient (amb), *press*, and *pulse*]) and in the water column of lake La Caldera. Data from 31 August represent concentrations before nutrient additions. n.m. represent not measured.

			DOC				
Day	Lake	UVR_{amb}	PAR_{amb}	UVR_{press}	PAR_{press}	UVR_{pulse}	PAR_{pulse}
31 August				49.16 (11.67)			
4 September	25.83 (1.67)	25.83 (1.67)	n.m.	52.5 (7.50)	41.67 (4.17)	46.67 (5.00)	65.00 (15.00)
9 September	70.00 (4.17)	25.83 (1.61)	44.17 (12.50)	71.67 (6.67)	73.33 (5.00)	62.50 (1.67)	79.17 (20.00)
12 September	n.m.	27.50 (2.50)	40.83 (5.00)	65.83 (7.50)	66.67 (9.17)	65.00 (3.33)	85.00 (7.50)
15 September	74.17 (11.67)	29.17 (1.67)	36.67 (5.83)	60.83 (3.33)	60.83 (3.33)	66.67 (9.17)	90.01 (0.00)
18 September	n.m.	29.17 (3.33)	31.67 (5.82)	110 (20.83)	67.50 (6.67)	49.17 (11.67)	111.67 (21.67)

Table S3: Results of two-way repeated-measures analysis of the variance (RM-ANOVA) of the interactive effects of ultraviolet radiation (UVR) and frequency of pulsed nutrients (P; treatments: ambient [amb], *press*, and *pulse*) on total dissolved phosphorus (TDP), total dissolved nitrogen (TDN) and dissolved organic carbon (DOC). *F* represents *F*-test values, numbers in bold indicate p < 0.05, and n.s. means not significant.

Variable	Treatment	*F*	p
TDP			
	UVR	21.95	**<0.001**
	P	0.96	n.s.
	Time	6.38	**<0.001**
	UVR × P	0.08	n.s.
	UVR × Time	12.82	**< 0.001**
	P × Time	2.93	**< 0.01**
	UVR × P × Time	2.17	**< 0.05**
TDN			
	UVR	30.01	**<0.001**
	P	38.13	**<0.001**
	Time	112.06	**< 0.001**
	UVR × P	2.87	n.s.
	UVR × Time	27.8	**< 0.001**
	P × Time	186.65	**< 0.001**
	UVR × P × Time	16.64	**< 0.001**
DOC			
	UVR	0.5	n.s.
	P	32.15	**<0.001**
	Time	59.4	**< 0.001**
	UVR × P	4.27	**< 0.05**
	UVR × Time	21.85	**< 0.001**
	P × Time	29.21	**< 0.001**
	UVR × P × Time	24.56	**< 0.001**

Table S4: Mean (± SD) of chlorophyll *a* (Chl *a*, in µg L^{-1}) concentrations measured during the incubation period in each experimental treatment (radiation [UVR, > 280 nm; PAR, > 400 nm] and nutrients [ambient (amb), *press*, and *pulse*]).

Day	UVR_{amb}	PAR_{amb}	UVR_{press}	PAR_{press}	UVR_{pulse}	PAR_{pulse}
31August			3.19 (0.41)			
4 September	3.89 (0.21)	4.37 (0.27)	12.52 (0.85)	13.61 (3.03)	13.54 (0.32)	17.91 (1.06)
9 September	3.91 (0.08)	2.08 (1.26)	26.72 (5.05)	25.6 (0.50)	25.02 (2.42)	25.36 (3.04)
12 September	3.85 (0.26)	1.84 (0.14)	11.48 (0.00)	10.51 (2.95)	4.36 (0.01)	15.62 (3.60)
15 September	4.53 (0.69)	2.14 (0.31)	12.55 (0.00)	11.32 (2.99)	5.33 (1.58)	3.38 (0.95)
18 September	2.96 (0.04)	1.69 (0.11)	23.03 (0.14)	10.33 (4.53)	13.78 (2.36)	4.99 (2.69)

Table S5: Results of two way-repeated measures analysis of variance (RM-ANOVA) of the interactive effect of ultraviolet radiation (UVR) and frequency of pulsed nutrients (P; treatments: ambient [amb], *press*, and *pulse*) on chlorophyll *a* (Chl *a*), total phytoplankton (PB) and bacteria biomass (BB). *F* represents *F*-test values, numbers in bold indicate p < 0.05, and n.s. means not significant.

Variable	Treatment	*F*	p
Chl *a*			
	UVR	0.13	n.s.
	P	130.29	**<0.001**
	Time	457.65	**< 0.001**
	UVR × P	0.92	n.s.
	UVR × Time	47.29	**< 0.001**
	P × Time	126.37	**< 0.001**
	UVR × P × Time	24.50	**< 0.001**
PB			
	UVR	3.24	n.s.
	P	58.23	**< 0.01**
	Time	160.66	**< 0.001**
	UVR × P	5.09	**< 0.05**
	UVR × Time	20.91	**< 0.001**
	P × Time	52.17	**< 0.001**
	UVR × P × Time	11.65	**< 0.001**
BB			
	UVR	16.25	**< 0.001**
	P	85.98	**< 0.001**
	Time	146.91	**< 0.001**
	UVR × P	0.07	n.s.
	UVR × Time	6.10	**< 0.001**
	P × Time	6.70	**< 0.001**
	UVR × P × Time	3.69	**< 0.001**

Table S6: Results of the non-linear fitting through cubic ($y = ax^3 + bx^2 + cx + d$) and peak-Gaussian functions ($y = y_0 + a \exp(- 0.5 ((x – b) / w^2))$) for mixotrophic nanoflagellates (MNFs) and photoautotrophs during the incubation period in each experimental treatment ([UVR, > 280 nm; PAR, > 400 nm] and nutrients [ambient (amb), *press*, and *pulse*]). R^2 represent the determination coefficient, numbers in bold indicate $p < 0.05$, and - means not-significant relationship.

	MNFs		Photoautotrophs	
Treatment	R^2	p	R^2	p
UVR_{amb}	-	-	0.90	**< 0.001**
PAR_{amb}	-	-	0.92	**< 0.001**
UVR_{press}	0.76	**< 0.001**	0.70	**< 0.01**
PAR_{press}	0.98	**< 0.001**	0.66	**< 0.001**
UVR_{pulse}	0.79	**< 0.001**	0.75	**< 0.001**
PAR_{pulse}	0.98	**< 0.001**	0.86	**< 0.001**

Table S7: Results of two-way analysis of variance (ANOVA) of the interactive effect of ultraviolet radiation (UVR) and intensity (ambient [amb], moderate [mod], and intense [int]) or frequency (ambient [amb], *press*, and *pulse*) of pulsed nutrients (P) on bacterial carbon demand : excretion of organic carbon ratio (BCD : EOC ratio). *F* represents *F*-test values, numbers in bold indicate p < 0.05, and n.s. means not significant.

	Intensity		Frequency	
Treatment	*F*	p	*F*	p
UVR	5.39	n.s.	0.36	n.s.
P	14.64	**<0.001**	10.22	**<0.01**
UVR × P	1.83	n.s.	3.97	**0.05**

Table S8: Results of two-way analysis of variance (ANOVA) of the interactive effect of ultraviolet radiation (UVR) and *intensity* of pulsed nutrients (P; treatments: ambient [amb], moderate [Mod], and intense [Int]) on sestonic P, sestonic carbon : phosphorus ratio (C:P), bacterial production (BP), primary production (PP), excretion of organic carbon (EOC), bacterial respiration (BR), bacterial growth efficiency (BGE), and gross assimilation of excreted photosynthetic carbon by bacteria (%PEGA). *F* represents *F*-test values, numbers in bold indicate $p < 0.05$, and n.s. means not significant.

Variable	Treatment	*F*	p
Sestonic P			
	UVR	182.03	**<0.001**
	P	3116.80	**<0.001**
	UVR × P	115.21	**<0.001**
C:P ratio			
	UVR	676.51	**<0.01**
	P	149.13	**<0.001**
	UVR × P	42.50	**<0.001**
BP			
	UVR	9.37	**<0.01**
	P	33.50	**<0.001**
	UVR × P	20.96	**<0.001**
PP			
	UVR	121.44	**<0.001**
	P	262.91	**<0.001**
	UVR × P	12.77	**<0.001**
EOC			
	UVR	54.02	**<0.001**
	P	27.06	**<0.001**
	UVR × P	0.47	n.s.
BR			
	UVR	0.01	n.s.
	P	26.13	**<0.001**
	UVR × P	25.66	**<0.001**
BGE			
	UVR	15.9	**<0.01**
	P	7.70	**<0.01**
	UVR × P	14.43	**<0.001**
%PEGA			
	UVR	0.97	n.s.
	P	4.64	**<0.05**
	UVR × P	1.93	n.s.

Table S9: Results of two-way analysis of variance (ANOVA) of the interactive effect of ultraviolet radiation (UVR) and frequency of pulsed nutrients (P; treatments: ambient [amb], *press*, and *pulse*) on sestonic P, sestonic carbon : phosphorus ratio (C:P), bacterial production (BP), primary production (PP), excretion of organic carbon (EOC), bacterial respiration (BR), bacterial growth efficiency (BGE) and gross assimilation of excreted photosynthetic carbon by bacteria (%PEGA). *F* represents *F*-test values, numbers in bold indicate $p < 0.05$, and n.s. means not significant.

Variable	Treatment	*F*	p
Sestonic P			
	UVR	212.93	**<0.001**
	P	634.05	**<0.001**
	UVR × P	36.16	**<0.001**
C:P ratio			
	UVR	18.19	**<0.001**
	P	88.13	**<0.001**
	UVR × P	33.12	**<0.001**
BP			
	UVR	6.5	**<0.05**
	P	20.89	**<0.001**
	UVR × P	15.64	**<0.001**
PP			
	UVR	7.74	**<0.05**
	P	189.36	**<0.001**
	UVR × P	7.62	**<0.01**
EOC			
	UVR	0.69	n.s.
	P	40.89	**<0.001**
	UVR × P	5.93	**<0.05**
BR			
	UVR	0.98	n.s.
	P	9.71	**<0.01**
	UVR × P	3.01	n.s.
BGE			
	UVR	0.03	n.s.
	P	4.72	**<0.05**
	UVR × P	16.7	**<0.001**
%PEGA			
	UVR	0.41	n.s.
	P	6.08	**<0.01**
	UVR × P	2.46	n.s.

Supplementary figures:

Figure S1. (A) Map of the study site showing a general view of lake La Caldera in Sierra Nevada National Park with the Mulhacen peak (3484 m.a.s.l.) in the background. (B) *In situ* disposition of the racks containing the experimental mesocosms and (C) detailed view of one of these structures into the lake. Map was created using Ocean Data View v.4.7.6 (http://odv.awi.de) and the photographs were taken by MJC.

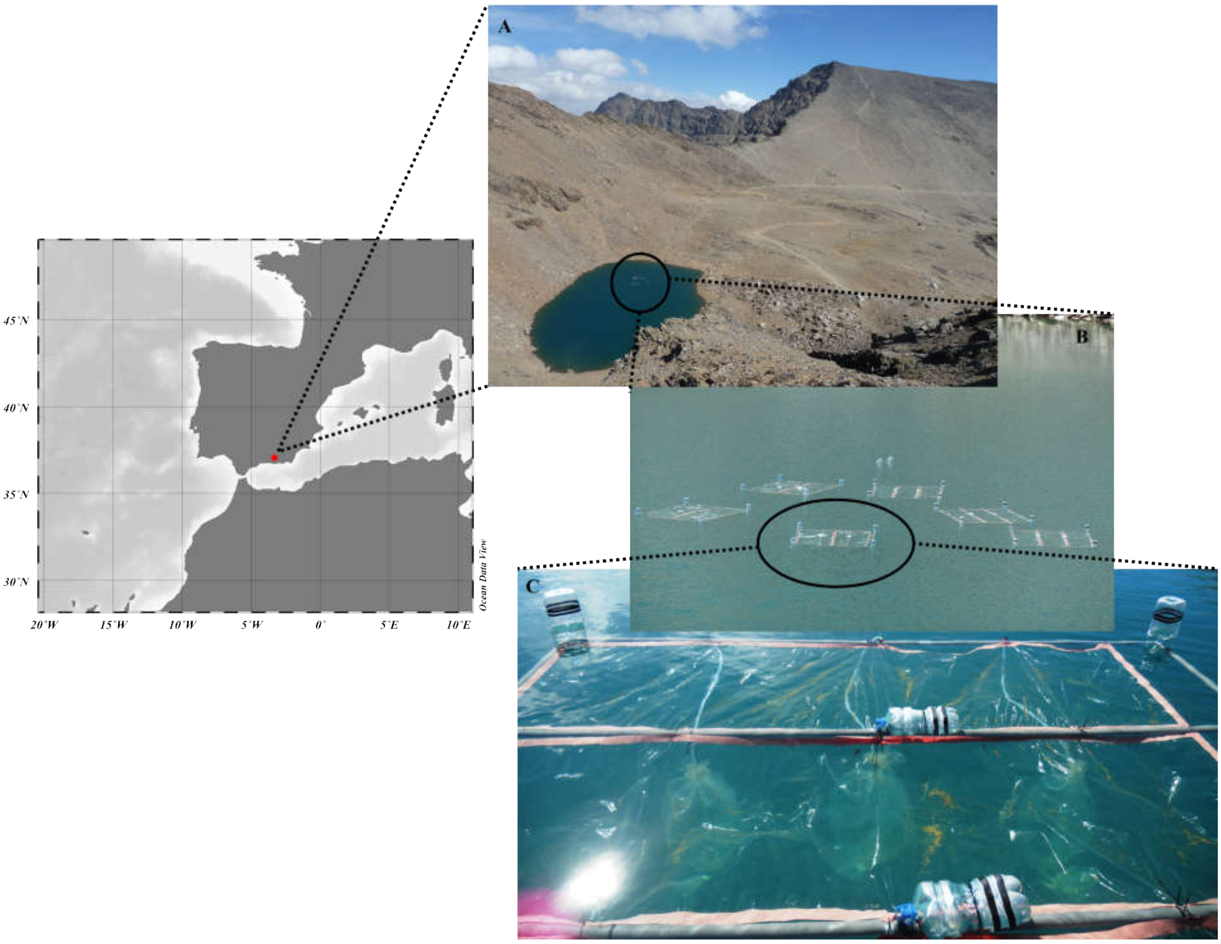

Figure S2. (A) Temperature and (B) solar irradiance as a function of depth in lake La Caldera. Irradiance data in the UVR portion are expressed in μW cm^{-2} nm^{-1}, PAR is in μmol photons m^{-1} s^{-1}.

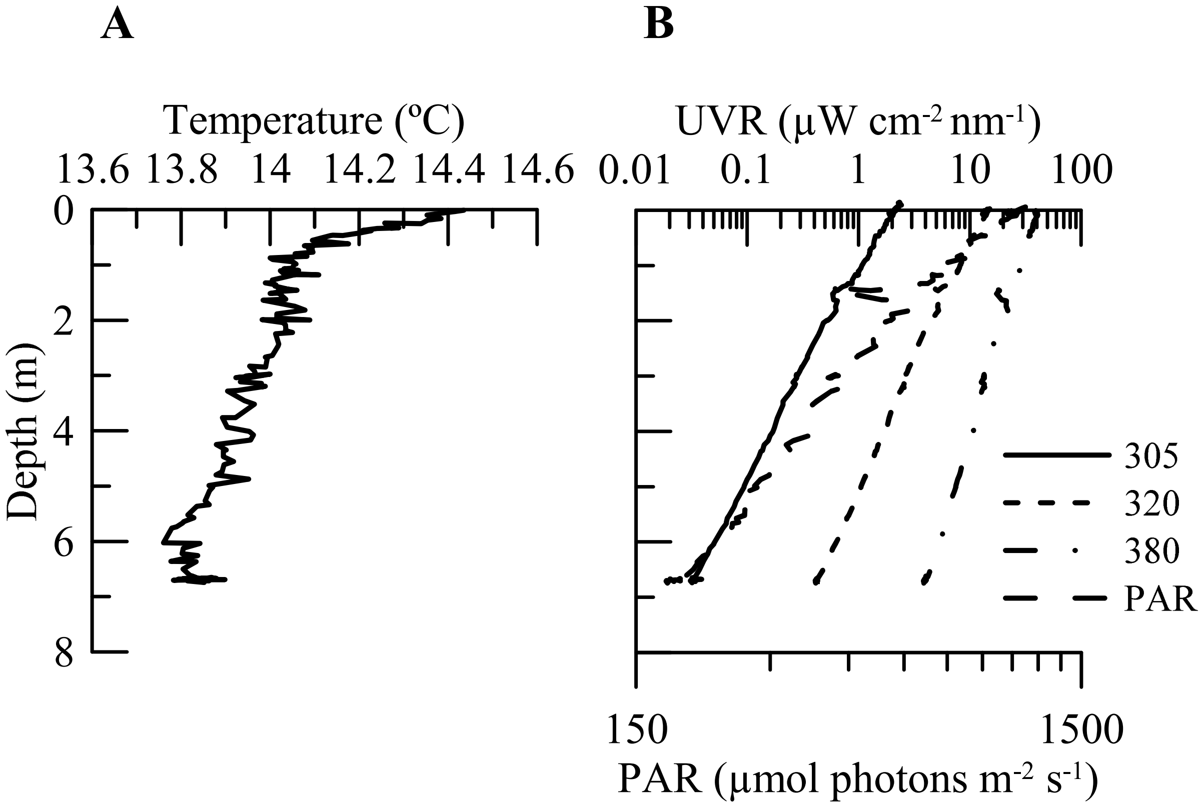

Figure S3. Mean (± SD) bacterial carbon demand : excretion of organic carbon (BCD : EOC) ratio under two radiation treatments, UVR (> 280 nm, white bars) and PAR (> 400 nm, black bars), and two pulsed nutrient regimes: (A) intensity, with ambient (amb), moderate (mod), and intense (int) pulse treatments; and (B) frequency, with ambient (amb), *press* and *pulse* treatments.

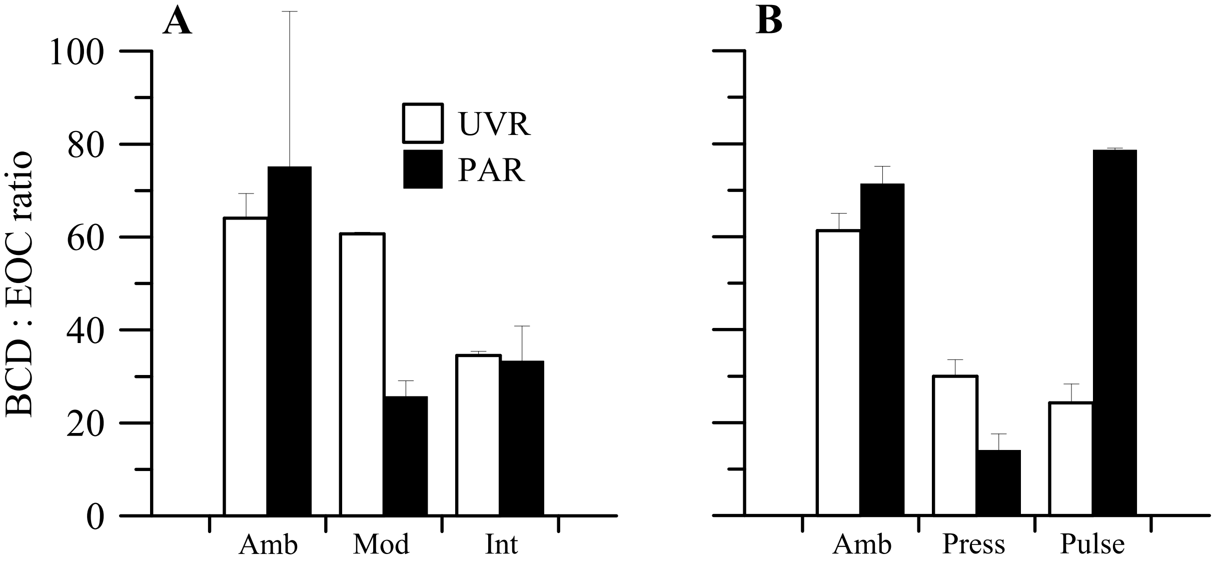

Figure S4. (A) Sestonic phosphorus (P) concentrations (in µg L^{-1}), (B) sestonic carbon : phosphorus ratio (C:P ratio), (C) bacterial production (BP, µg C L^{-1} h^{-1}), (D) primary production (PP, µg C L^{-1} h^{-1}), (E) excretion of organic carbon (EOC, µg C L^{-1} h^{-1}), (F) bacterial respiration (BR, < 0.7 µm; µM O_2 h^{-1}), (G) bacterial growth efficiency (BGE) and (H) percentage of photosynthetic excreted gross assimilation (%PEGA) in lake La Caldera under the two radiation treatments: UVR (white bars) and PAR (black bars) and the three nutrient treatments: ambient (amb), moderate (mod) and intense (int). The bars represent mean values of three replicates and lines on top of the bars are the standard deviation. Letters indicate differences among treatments by Bonferroni *post hoc* test. Note that these results represent responses observed over the short term when communities received different amounts of nutrients (Mod, 5 µg P L^{-1} and Int, 30 g P L^{-1}).

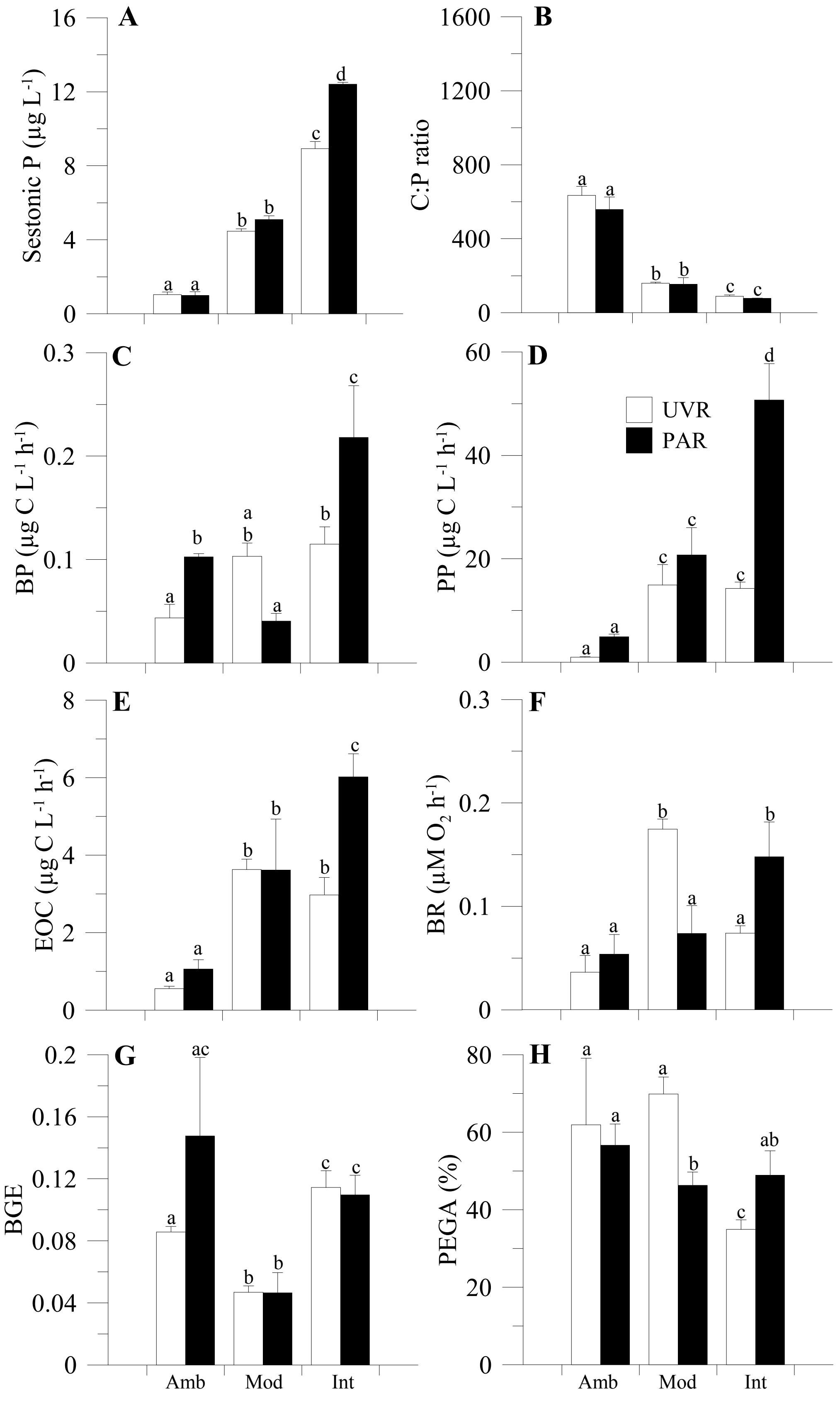
A
B
C
D
E
F
G
H
Sestonic P (µg L-1)
C:P ratio
BP (µg C L-1 h-1)
PP (µg C L-1 h-1)
EOC (µg C L-1 h-1)
BR (µM O2 h-1)
BGE
PEGA (%)
UVR
PAR
Amb
Mod
Int

Supplementary references

1 APHA. *Standard methods for the examination of water and wastewater*. (American Public Health Association, 1992).

2 Benner, R. & Strom, M. A critical evaluation of the analytical blank asociated with DOC measurements by high-temperature catalytic oxidation. *Mar. Chem.* **41**, 153-160 (1993).

3 Tanaka, J. S. How big is enough? sample size and goodness of fit in structural equation models with latent variables. *Child Dev.***58**, 134-146 (1987).

4 Zar, J. H. *Biostatistical analysis*. 4th edn (Prentice Hall, 1999).

5 Iriondo, J. M., Albert, M. J. & Escudero, A. Structural equation modelling: An alternative for assessing causal relationships in threatened plant populations. *Biol. Conserv.* **113**, 367-377 (2003).

6 Lohelin, J. C. *Latent variable models. An introduction to factor, path and strutural analysis* (Lawrence Erlbaum Associates, 1992).

7 Bullejos, F. J., Carrillo, P., Villar-Argaiz, M. & Medina-Sánchez, J. M. Roles of phosphorus and ultraviolet radiation in the strength of phytoplankton–zooplankton coupling in a Mediterranean high mountain lake. *Limnol. Oceanogr.* **55**, 2549–2562 (2010).

8 Morales-Baquero, R., Pulido-Villena, E. & Reche, I. Atmospheric inputs of phosphorus and nitrogen to the southwest Mediterranean region: Biogeochemical responses of high mountain lakes. *Limnol. Oceanogr.* **51**, 830-837 (2006).

9 Acker, J. G. & Leptoukh, G. Online analysis enhance NASA Earth science data. *EOS, Trans. AGU* **88**, 14-17 (2007).

10 Carrillo, P., Reche, I., Sánchez-Castillo, P. & Cruz-Pizarro, L. Direct and indirect effects of grazing on the phytoplankton seasonal succession in an oligotrophic lake. *J. Plankton Res.* **17**, 1363-1379 (1995).

11 Rocha, O. & Duncan, A. The relationship between cell carbon and cell volume in freshwater algal species used in zooplankton studies. *J. Plankton Res.* **7**, 279-294 (1985).

12 Gasol, J. M. & Del Giorgio, P. Using flow cytometry for counting natural planktonic bacteria and understanding the structure of planktonic bacterial communities. *Sci. Mar.* **64**, 197-224 (2000).

13 Zubkov, M. V., Burkill, P. H. & Topping, J. N. Flow cytometric enumeration of DNA-stained oceanic planktonic protists. *J. Plankton Res.* **29**, 79-86 (2007).

14 Zubkov, M. V. & Burkill, P. H. Syringe pumped high speed flow cytometry of oceanic phytoplankton. *Cytom. A* **69**, 1010-1019 (2006).

15 Posch, T. *et al.* Precision of bacterioplankton biomass determination: a comparison of two fluorescent dyes, and of allometric and linear volume-to-carbon conversion factors *Aquat. Microb. Ecol.* **25**, 55-63 (2001).

16 Dorado-García, I., Medina-Sánchez, J. M., Herrera, G., Cabrerizo, M. J. & Carrillo, P. Quantification of carbon and phosphorus co-limitation in bacterioplankton: New insights on an old topic. *PLoS One* **9**, e99288 (2014).

17 Carrillo, P., Medina-Sánchez, J. M. & Villar-Argaiz, M. The interaction of phytoplankton and bacteria in a high mountain lake: Importance of the spectral composition of solar radiation. *Limnol. Oceanogr.* **47**, 1294-1306 (2002).

18 Medina-Sánchez, J. M., Villar-Argaiz, M. & Carrillo, P. Solar radiation - nutrient interaction enhances the resource and predation algal control on bacterioplankton: A short-term experimental study. *Limnol. Oceanogr.* **51**, 913-924 (2006).

19 Bell, R. T. in *Handbook of methods in aquatic microbial ecology* (eds P. F. Kemp, B. F. Sherr, & J. J. Cole) 495-503 (Lewis Publishers, 1993).

20 Lee, S. & Fuhrman, J. A. Relationships between biovolume and biomass of naturally derived marine bacterioplankton. *Appl. Environ. Microbiol.* **53**, 1298-1303 (1987).
